# Infrared UAV Target Detection Based on Continuous-Coupled Neural Network

**DOI:** 10.3390/mi14112113

**Published:** 2023-11-18

**Authors:** Zhuoran Yang, Jing Lian, Jizhao Liu

**Affiliations:** 1 School of Information Science and Engineering, Lanzhou University, Lanzhou 730000, China; yangzhr2021@lzu.edu.cn; 2 School of Electronics and Information Engineering, Lanzhou Jiaotong University, Lanzhou 730070, China; lian322scc@163.com

**Keywords:** CCNN, infrared image processing, UAV detection

## Abstract

The task of the detection of unmanned aerial vehicles (UAVs) is of great significance to social communication security. Infrared detection technology has the advantage of not being interfered with by environmental and other factors and can detect UAVs in complex environments. Since infrared detection equipment is expensive and data collection is difficult, there are few existing UAV-based infrared images, making it difficult to train deep neural networks; in addition, there are background clutter and noise in infrared images, such as heavy clouds, buildings, etc. The signal-to-clutter ratio is low, and the signal-to-noise ratio is low. Therefore, it is difficult to achieve the UAV detection task using traditional methods. The above challenges make infrared UAV detection a difficult task. In order to solve the above problems, this work drew upon the visual processing mechanism of the human brain to propose an effective framework for UAV detection in infrared images. The framework first determines the relevant parameters of the continuous-coupled neural network (CCNN) through the image’s standard deviation, mean, etc. Then, it inputs the image into the CCNN, groups the pixels through iteration, then obtains the segmentation result through expansion and erosion, and finally, obtains the final result through the minimum circumscribed rectangle. The experimental results showed that, compared with the existing most-advanced brain-inspired image-understanding methods, this framework has the best intersection over union (IoU) (the intersection over union is the overlapping area between the predicted segmentation and the label divided by the joint area between the predicted segmentation and the label) in UAV infrared images, with an average of 74.79% (up to 97.01%), and can effectively realize the task of UAV detection.

## 1. Introduction

In recent years, unmanned aerial vehicles (UAVs) have been widely used in various civilian and commercial applications. Due to the endless non-cooperative intrusion incidents of civilian UAVs in low-altitude areas, UAV target detection technology has received widespread attention. Initially, UAV detection methods mainly included the following types: radar detection, radio frequency detection, photoelectric identification tracking, and sound monitoring. However, the electromagnetic signals emitted by small UAVs are very limited [1,2], difficult to detect with conventional radar, and do not handle the Doppler effect well [3]. With the development of artificial intelligence in image-processing technology, image-recognition methods have made significant progress in the detection of UAVs. The above method requires manually constructing a feature extractor based on the shape, color, and other characteristics of the identified target to extract information, which is greatly affected by the background and has high environmental requirements. However, in reality, UAVs often need to be identified in complex environments such as at night or in rainy and snowy weather. Small UAVs are not easily captured by cameras [4,5], resulting in unclear features. The above method cannot operate in this environment.

Infrared detection technology has the advantage of not being interfered with by environmental factors, so it has a wide application scenario. It can collect targets in special environments such as at night. Compared with optical images, detecting UAVs with infrared images has many advantages like better thermal sensitivity and all-weather detection capability [6,7]. For example, infrared imaging receives infrared radiation; it does not require high lighting conditions and can be used at night. It has a strong reconnaissance ability because infrared radiation has a thermal effect and can pass through clouds. Infrared imaging can observe targets hidden behind clouds, independent of weather changes [8]. Therefore, using images collected by infrared thermal imaging instruments instead of color images has become the main method to realize target detection in special environments such as at night.

**Figure 1:**
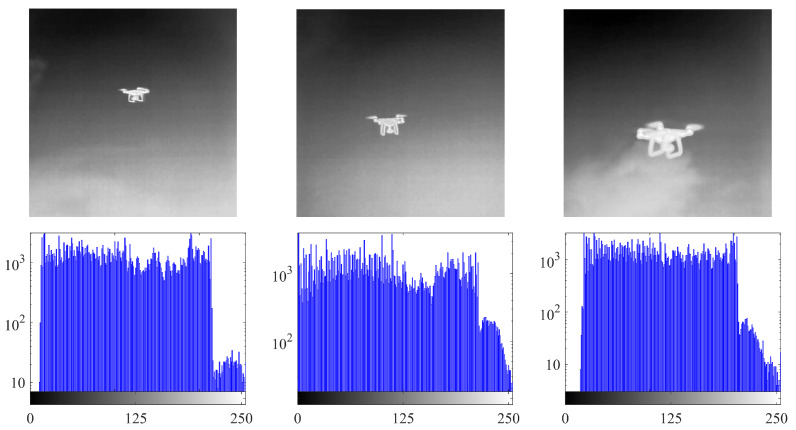
Infrared image and its histogram.

It is difficult to identify UAVs in infrared images using the most-advanced deep learning and underlying image-processing methods. This is because of the following: (1) Since infrared detection equipment is expensive and data collection is difficult, existing methods have difficulty detecting UAVs. There are few infrared images of these machines, making it difficult to train deep neural networks. (2) In addition, there are background clutter and noise in infrared images, such as heavy clouds and buildings, thus a low signal-to-clutter ratio and a low signal-to-noise ratio [9]. It is difficult to achieve the task of UAV detection using traditional methods. As shown in Figure 1, when there is cloud interference, the histogram of the infrared image will not have an obvious bimodal shape or a nearly bimodal, unimodal, or multimodal shape.

To solve the above problems, this work drew inspiration from the visual processing mechanisms of mammals and introduces a processing mechanism that simulates the visual cortex of the human brain into the infrared image processing. The CCNN neurons encode the grayscale values of the pixels in the image as the firing frequency of the neurons [10]. The identification of the target objects is achieved by distinguishing the frequencies corresponding to neural clusters. The higher the grayscale value of a pixel, the higher the frequency of the output signal. If the grayscale values of the pixels are similar, their frequencies are also close. This image-processing method can reduce the local gray level difference of the image and compensate for small local discontinuities in the image. However, there is currently no existing method based on the CCNN for the automatic processing of infrared images. In this work, a framework for automatically detecting UAVs in infrared images is proposed. The framework first sets the relevant parameters of the continuous-coupled neural network (CCNN) through the standard deviation, mean, etc., of the image. Then, it inputs the image into the CCNN, groups the pixels through iterations, uses the entropy of the image as an indicator to control the number of iterations, then obtains the segmentation results through expansion and erosion, and finally, obtains the detection results of the UAV through the minimum circumscribed rectangle. The framework can automatically identify UAVs in infrared images and overcome the difficulty of deep learning due to the small number of samples.

In summary, the main contributions of this work are listed as follows:

(1) It introduces the primary visual cortex model into infrared image processing and analyzes the status characteristics of the CCNN in the infrared image processing.
(2) It proposes a new framework for the automatic detection of infrared UAVs. This framework is capable of automatically configuring the parameters based on the input image, then groups the image pixel values through the iterative process of the CCNN, reconstructs the image through the output value, and controls the number of iterations through the entropy of the image.
(3) The proposed detection framework was tested in a complex environment to verify the effectiveness of the method. This work was performed on infrared images collected with complex buildings and cloud cover. The average IoU of this framework in the UAV infrared images reached 74.79% (up to 97.01%), which can effectively achieve unmanned operation for machine detection tasks.

## 2. Related Works

### 2.1. UAV Detection Method Based on Machine Learning

With the development of artificial intelligence in image-processing technology, many scholars have made significant progress in the research on UAV detection. Molchanov et al. extracted features based on the eigenvectors and eigenvalues of multi-dimensional scaling (MDS) (a classic dimensionality reduction method) [11,12]. They trained a linear and a non-linear support vector machine (SVM) (a support vector machine is a two-class classification model, and its basic model is a linear classifier defined with the largest interval in the feature space), as well as a naive Bayes classifier. In a second test, the authors excluded some models from the training and found that the classifier could still classify them into fixed-wing, stationary rotor, or helicopter with an accuracy ranging from 87% to 100%. The challenge was a robust alignment of the m-D signature. The proposed feature-extraction algorithm requires the m-D signature to be properly aligned. The current alignment method does not assume discontinues in the m-D signature (e.g., when a part of the signature is shadowed by another object). For such scenarios, a more-robust alignment procedure is required [11]. Jahangir and Baker trained a binary decision tree model, which could improve the UAV prediction probability to 88% and reduce the false alarm rate to 0 [13,14]. But, the model was trained based on the Doppler effect of the UAVs; the electromagnetic signals emitted by small UAVs are very limited [1,2], difficult to detect with conventional radar, and do not handle the Doppler effect well [3]. Therefore, this method still has limitations for UAV detection in special environments. Olusiji et al. used various categories of wavelet transforms (discrete wavelet transform, continuous wavelet transform, and wavelet scattering transform) to extract features from the signals to build models using these features. They used the wavelet scattering transform to extract signatures (scattergrams) from the steady state of the radio frequency (RF) signals at a 30 dB signal-to-noise ratio (SNR) and used these scattergrams to train the SqueezeNet. They achieved an accuracy of 98.9% at a 10 dB SNR [15]. However, using various categories of wavelet transforms for extracting features from the signals is a complicated task, and the electrical signals radiated by UAVs are not obvious in some complex environments, which brings great challenges to the model construction.

However, the above method needs to manually construct a feature extractor to extract information according to the shape, color, and other characteristics of the target. Feature extraction is one of the more-difficult steps in image pattern recognition. Some sources of difficulty are the presence of irrelevant information and the relationshipof a feature set to a particular application [16]. This requires high professional knowledge and is prone to problems such as poor generalization ability and difficulty in achieving real-time detection efficiency. The classifier structure is more difficult to interpret, which tends to worsenwith the number of features. Further, the prediction variability tends to increase, and the classifier is sensitive to outliers. There is no guarantee that the classifier will perform as well on a new set of samples [17].

### 2.2. UAV Detection Method Based on Deep Learning

Deep learning has shown great promise in computer vision and pattern recognition [18,19], and deep neural networks are increasingly used for image segmentation [20] and image fusion [21]. Deep learning is, therefore, a promising means of detecting and identifying UAVs [18,22]. In [18], a YOLOv2-based (YOLO is a target detection model. Its full name is you only look once. This means that you only need to browse once to identify the category and location of the objects in the picture.) object-detection algorithm was chosen for loaded and unloaded UAV detection, and the average precision reached 74.94%. This was the first time a YOLOv2-based algorithm was introduced to loaded and unloaded UAV object detection. However, the data scene collected by this method is relatively simple, and there are no other interference factors such as noise. It still needs to be verified on more datasets [18]. Wang et al. proposed a convolutional neural network (CNN) with two heads [23]: one for the classification of the input range Doppler map patch into the target being present or absent and the other for the regression of the offset between the target and the patch center. Then, based on the output of the network, a nonmaximum suppression (NMS) mechanism composed of probability-based initial recognition, distribution-density-based recognition, and voting-based regression was developed to reduce false alarms, as well as control the false alarms. Finally, experiments on both simulated data and real data were carried out, and it was shown that the proposed method could locate the target more accurately and achieve a much lower false alarm rate at a comparable detection rate than the constant false alarm rate (CFAR). Due to the input of the network actually being a series of patches obtained from the R-D map, the training and testing process of the proposed method was rather time-consuming, which limits its application in real life. In addition to the above methods, algorithms [1,24] such as R-CNN, FastR-CNN, and FasterR-CNN are also widely used in UAV detection.

However, the above work was all based on the training and testing of images collected by visible light cameras, and it did not take into account the situation that the optical lens cannot capture the UAVs in a complex environment. The above method has limited room for improvement in detecting UAVs in clouds at night. Although infrared images have the advantage of not being interfered with by environmental factors, infrared detection equipment is relatively expensive and data collection is difficult, so there are few existing infrared images based on UAVs, making it difficult to train deep neural networks based on infrared images.

### 2.3. Brain-Inspired Computing

There are billions of neurons in the human brain. The dendrite is the input terminal [25]. The cell body integrates incoming spikes received by different branches of the dendrites and generates a spike when its membrane potential reaches the threshold. Spikes travel along the axons to other neurons via synapses [26,27,28].

Various types of brain-inspired models have been proposed to faithfully simulate the human brain. In recent years, spiking neural networks (SNNs) have attracted enormous research interest. There has been an upward trend in SNN-related papers [25]. For example, a variety of spiking neuron models have been proposed [10] to emulate the generation of spikes with different levels of fidelity and computational cost; many memristor-based models have been proposed [29,30] to simulate synapses [31].

More and more brain-inspired models have been used in the field of computer vision. The pulse-coupled neural network (PCNN) is the third-generation artificial neural network proposed in the 1990s. Its network is constructed by simulating the activity of mammalian visual cortex neurons. It is also called the third-generation neural network. Since the PCNN model was developed based on the study of mammalian physiological and visual characteristics, it has obvious advantages in target segmentation [32].

Inspired by the primary visual cortex of mammals, such as cats, PCNN models can display synchronized oscillations and process digital images without training. Over the past few decades, many studies [33,34,35] have demonstrated that PCNN models have better performance [36,37] than other neuron models in image-processing tasks. Di et al. [36] performed medical image fusion based on NSCT domain rolling guidance filtering and an adaptive PCNN. Qi et al. [37] performed breast density segmentation based on the Morph SPCNN model. However, PCNN models exhibit periodic behavior under a periodic stimulus [10]. This is the main obstacle for PCNNs to create a brain-like machine.

The continuous-coupled neural network (CCNN) is a new brain-like neural network. It was evolved from the PCNN. The difference is that the discharge process in all PCNN models is deterministic and cannot realistically simulate human brain neurons. The CCNN can show periodic behavior under constant stimulation, as well as chaotic behavior [38] under periodic stimulation and can simulate human brain neurons more realistically [10].

However, there is currently no method for processing infrared images based on the CCNN, and the CCNN requires manual adjustment of its parameters during image processing. There is no automatic image-segmentation solution based on the CCNN, which limits the application of the CCNN, especially for infrared images.

## 3. Method

In this section, we propose in detail a framework for UAV detection in infrared images that draws on the visual processing mechanisms of the human brain.

### 3.1. Continuous-Coupled Neural Network

Different from traditional artificial neural networks, the CCNN does not require any training and only has a single layer of laterally linked continuous-coupled neurons [39], which mainly includes five crucial components: couple linking, feeding input, modulation product, dynamic activity, and continuous output [32].

On the dendritic tree, the feeding synapses receive the external stimulus, which is the main input signal, and the action potential of the neighboring neurons. Moreover, the linking synapses are only associated with their adjacent neurons [32]. The interaction of the above two synapses produces the membrane potential of the neuron, which is compared with the dynamic threshold potential to judge whether the action potential is generated or not.

The CCNN is described as follows:

(1) $$\left\{\begin{array}{l} F_{ij}\left(n\right)=e^{-\alpha_{f}}F_{ij}\left(n-1\right)+V_{F}M_{ijkl}Y_{kl}\left(n-1\right)+S_{ij}\\ L_{ij}\left(n\right)=e^{-\alpha_{l}}L_{ij}\left(n-1\right)+V_{L}W_{ijkl}Y_{kl}\left(n-1\right)\\ U_{ij}\left(n\right)=e^{-\alpha_{f}}U_{ij}\left(n-1\right)+F_{ij}\left(n\right)\left(1+\beta L_{ij}\left(n\right)\right)\\ Y_{ij}\left(n\right)=\frac{1}{1+e^{-(U_{ij}\left(n\right)-E_{ij}\left(n\right))}}\\ E_{ij}\left(n\right)=e^{-\alpha_{e}}E_{ij}\left(n-1\right)+V_{E}Y_{ij}\left(n-1\right) \end{array}\right.$$

where

(2) $$W_{ijkl}=\left[\begin{array}{ccc} 0.5 & 1 & 0.5\\ 1 & 0 & 1\\ 0.5 & 1 & 0.5 \end{array}\right]$$

The five main parts are couple linking $L_{ij}$(*n*), feeding input $F_{ij}$(*n*), modulation product $U_{ij}$(*n*), dynamic activity $E_{ij}$(*n*), and continuous output $Y_{ij}$(*n*). The quantity $S_{ij}$ is the external feeding input received by the receptive fields. The parameters $\alpha_{f}$ and $\alpha_{e}$ denote exponential decay factors that record previous input states. The functions $V_{F}$ and $V_{L}$ are weighting factors modulating the action potentials of the surrounding neurons. Among them, $W_{ijkl}$ denote the feeding and linking synaptic weights, respectively, and β denotes the linking strength, which directly determines $L_{ij}$(*n*) in the modulation product $U_{ij}$(*n*).

**Figure 2:**
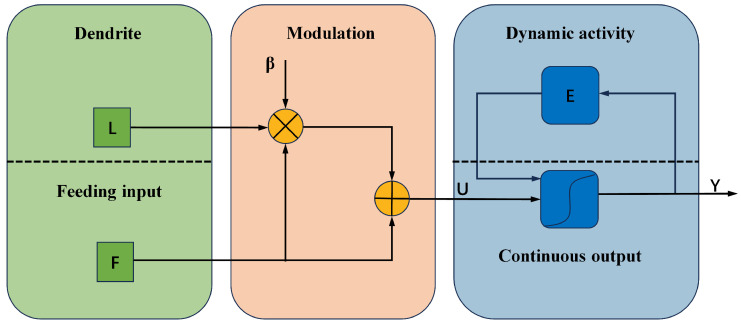
Continuous-coupled neuron network.

Figure 2 presents an intuitive illustration of the five parts.

The CCNN model can be considered as a self-organized method, i.e., the network is provided with inputs, but without the demand of the desired output. For image segmentation, the output of the CCNN often does not correspond well with the content of the images, because of the inner parameter setting mechanisms for adjusting the behavior of the neurons [40].

Based on Zhan et al. [41] and Chen et al.’s [42] research, we propose an automatic parameter-setting method for the CCNN tailored to infrared images. There are five adjustable parameters, $\alpha_{f}$, $\alpha_{e}$, β, $V_{E}$, and $V_{L}$, and these parameters can be set automatically.

(3) $$\left\{\begin{array}{l} \alpha_{f}=\alpha_{l}=\log_{}\frac{1}{\delta }\\ \beta =(\frac{1.5S_{max}/S^{{}^{\prime }}-1}{6V_{L}})\\ V_{E}=e^{-\alpha_{f}}+1+6\beta V_{L}\\ V_{L}=1\\ \alpha_{e}=\ln_{}\left(\frac{\frac{V_{E}}{S^{{}^{\prime }}}}{\frac{1-e^{-3\alpha_{f}}}{1-e^{-\alpha_{f}}}+6\beta V_{L}e^{-\alpha_{f}}}\right) \end{array}\right.$$

where $S^{{}^{\prime }}$ and $S_{max}$ denote the Otsu thresholding and the maximum intensity of the image. δ represents the standard deviation of the image. In the above equation, parameters β, $\alpha_{e}$, and $V_{E}$ always have a large impact on the CCNN. Obviously, the larger the value of β, the more strongly a neuron is influenced by its eight adjacent outputs. The larger the values of $\alpha_{e}$ or $V_{E}$, the lower the segmentation accuracy rates become.

### 3.2. Image-Processing Framework

**Figure 3:**
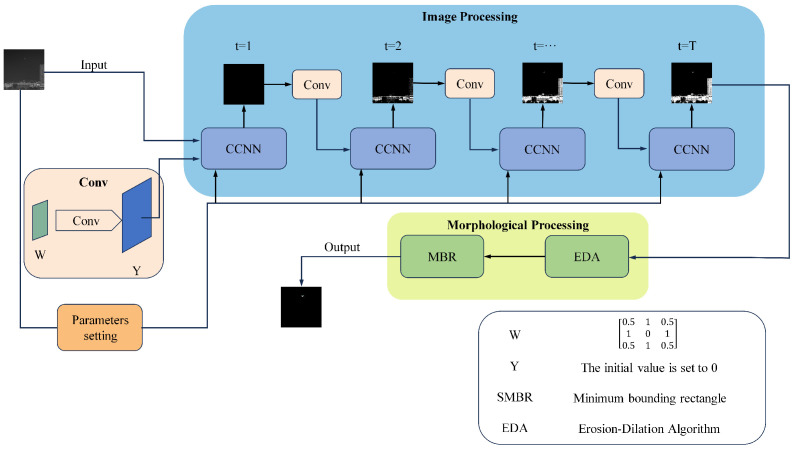
Image-processing framework of UAVs in infrared images.

Figure 3 below shows the basic framework of our use of the CCNN model to segment UAVs in infrared images. The CCNN model, which simulates the visual cortex of the human brain with simplified parameters, is used to process the infrared images, and morphological algorithms such as dilation and erosion are used to remove the background and successfully segment the UAV in the infrared image.

#### 3.2.1. Erosion–Dilation Algorithm

During the erosion operation, the minimum value of the values in the rectangular neighborhood of each position is taken as the output gray value of that position. The overall brightness of the output image after erosionis lower than that of the original image [43]. The area of the brighter areas in the image will become smaller, and the area of the darker areas will increase. Dilation is equivalent to the reverse operation of erosion, where lighter objects in the image will become larger and darker objects will decrease in size.

#### 3.2.2. Minimum Bounding Rectangle

After obtaining the segmentation result, the final result is obtained through the minimum bounding rectangle (MBR). The specific operation is to determine the UAV in the segmented image through the maximum connectivity [44] and, then, calculate the centroid to determine the bounding box.

## 4. Experiment

In this section, we conducted experiments on infrared images and compared them with the most-advanced underlying image-processing methods.

### 4.1. Dataset

First, we used a handheld infrared thermal-imaging device to collect some UAV data at night. The equipment parameters were as follows:

The IR resolution was 640 × 512 px; the pixel size was 12 μm; the focal length of the infrared objective lens was 50 mm; the field of view (FOV) was $8^{\circ }\times 6^{\circ }$; the focal length adaptation range was 50 m ∼∞.

**Figure 4:**
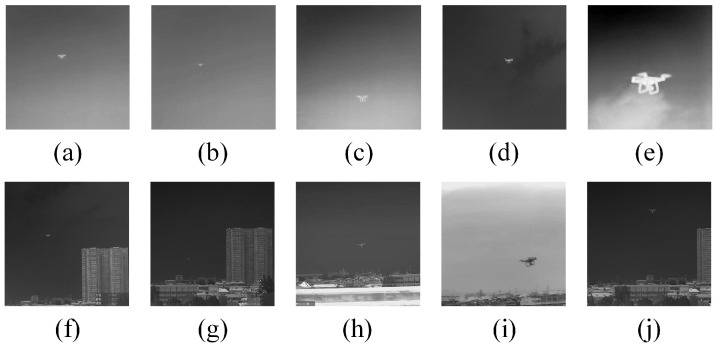
High-flying-height images: (**a**) Image 1, (**b**) Image 2, (**c**) Image 3, (**d**) Image 4, and (**e**) Image 5. Low-flying-height images: (**f**) Image 6, (**g**) Image 7, (**h**) Image 8, (**i**) Image 9, and (**j**) Image 10.

We collected a total of 19 UAV infrared images, then these data were divided into two categories: high-altitude UAV and low-altitude UAV with buildings in the background (Figure 4; Image 1∼5 were taken when the UAV was flying at a high altitude; Image 6∼10 were taken when the UAV was flying at a low altitude), and all the data were labeled using manual annotation. Figure 4 is a depiction of five samples from each of the two categories of datasets. The parameter settings of the experiment were the same as explained in Section 3.

### 4.2. Experimental Results

The results obtained by this model were then compared with those obtained by advanced underlying image-processing methods such as the PCNN, Otsu, and FCM.

**Figure 5:**
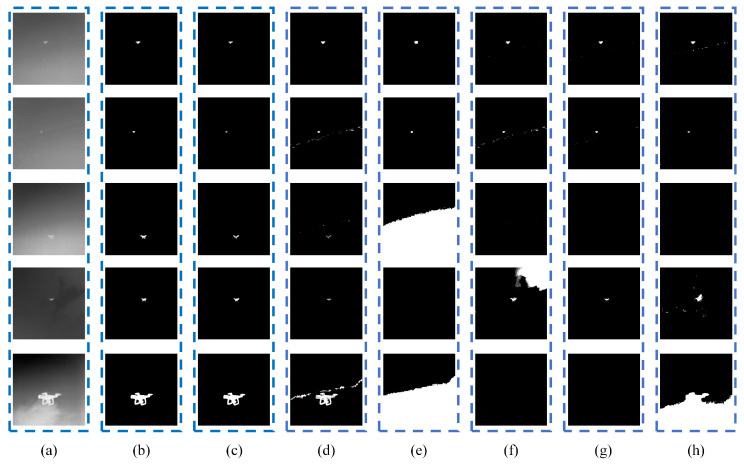
Comparative results obtained by different methods when the background is clouds: (**a**) an original image, (**b**) results of manual labeling, (**c**) results of the CCNN, (**d**) results of the PCNN, (**e**) results of FCM, (**f**) results of Otsu, (**g**) results of iteration, and (**h**) results of 2-mode.

The Figure 5 shows the set of experiments which was completed with data at a high flight altitude. At this time, the background interference factor was clouds.

**Figure 6:**
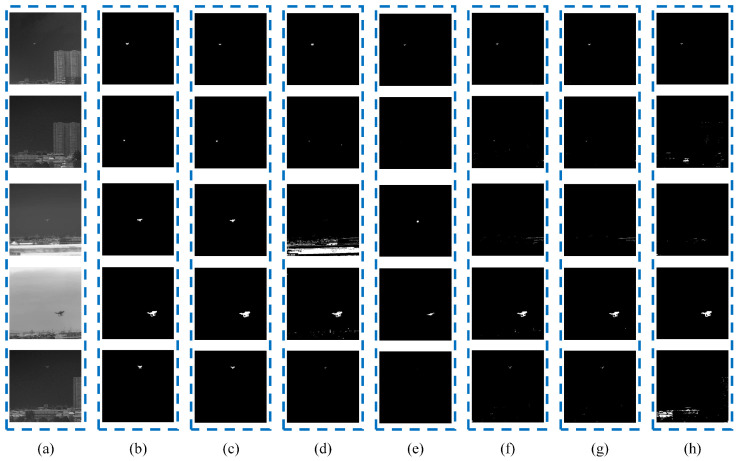
Comparative results obtained by different methods when the background is buildings: (**a**) an original image, (**b**) results of manual labeling, (**c**) results of the CCNN, (**d**) results of the PCNN, (**e**) results of FCM, (**f**) results of Otsu, (**g**) results of iteration, and (**h**) results of 2-mode.

The Figure 6 shows the set of experiments which was completed with data at a low flight altitude. At this time, the background interference factors were buildings.

**Figure 7:**
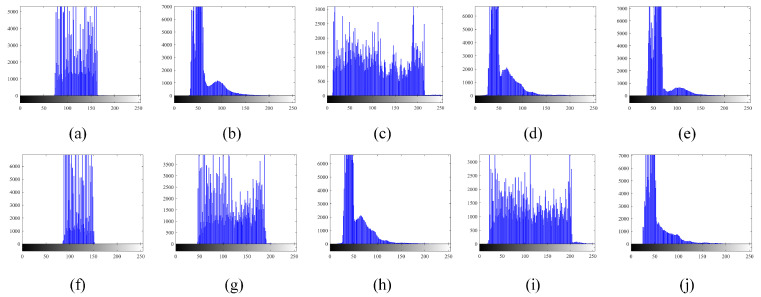
Histograms of experimental images: (**a**) Image 1, (**b**) Image 2, (**c**) Image 3, (**d**) Image 4, (**e**) Image 5, (**f**) Image 6, (**g**) Image 7, (**h**) Image 8, (**i**) Image 9, and (**j**) Image 10.

Figure 7 illustrates the corresponding histograms of the original infrared images. Although it can be observed that the shapes of the histograms are irregularly distributed, including bimodal or nearly bimodal, unimodal, and multimodal, our proposed model could effectively extract the targets from the background, as shown in Figure 5 and Figure 6.

### 4.3. Performance Measure

Considering that this research needed to identify UAVs in complex environments, we used the intersection over union (IoU) [45] of the images to evaluate the performance of the segmentation method. The IoU is the overlap area between the predicted segmentation and label divided by the joint area between the predicted segmentation and label (the intersection of the two/the union of the two). This metric ranges from 0∼1 (0∼100%), where 0 means no overlap and 1 means completely overlapping segmentations. The calculation formula is:

(4) $$IoU=\frac{\left|A\cap B\right|}{\left|A\cup B\right|}=\frac{TP}{TP+FP+FN}$$

In the segmentation task, the samples can be divided into true examples (true positive (*TP*), false positive (*FP*) examples, true negative (*TN*) examples, and false negative (*FN*) examples. *TP*, *FP*, and *FN*, respectively, represent the number of corresponding samples.

Table 1 lists the performance metrics for each algorithm. As can be seen, the results of our model seemed to be the best, with the highest IoU value. This proved that our model outperformed the above methods in terms of segmentation performance.

After conducting the tests on all the data, we found that the average IoU of the CCNN was the highest, reaching 74%; the PCNN, iteration, Otsu, and bimodal methods showed similar performance, with scores of 46.96%, 41.11%, 40.61%, and 35.50% respectively; FCM performed the poorest, with a score of 22.56%.

### 4.4. Detection Results

**Figure 8:**
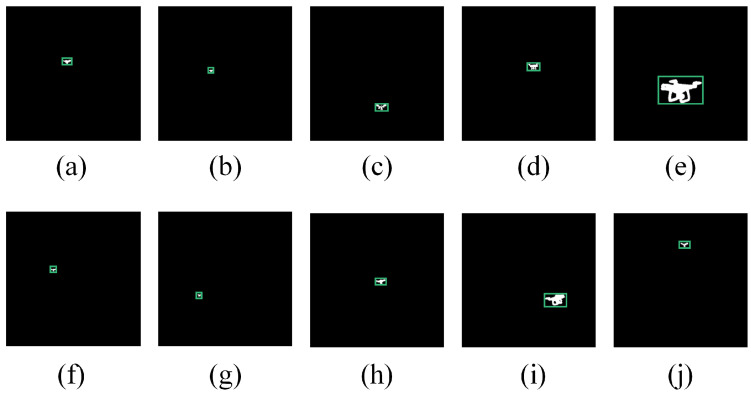
Final results: (**a**) Image 1, (**b**) Image 2, (**c**) Image 3, (**d**) Image 4, (**e**) Image 5, (**f**) Image 6, (**g**) Image 7, (**h**) Image 8, (**i**) Image 9, and (**j**) Image 10.

After obtaining the segmentation results, we used the erosion and expansion and minimum enclosing rectangle algorithms to obtain the detection results. The result are shown in Figure 8.

### 4.5. Status Characteristics

**Figure 9:**
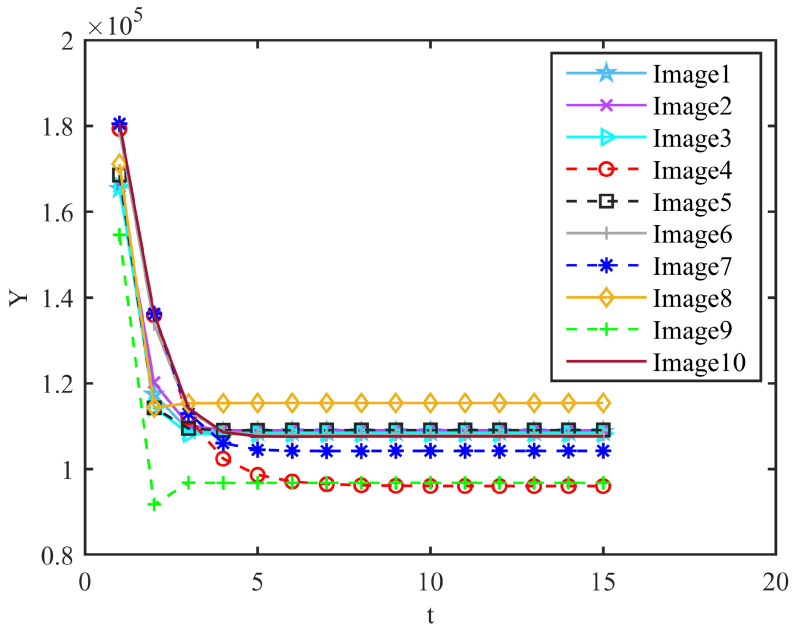
Value of Y during CCNN iteration.

**Figure 10:**
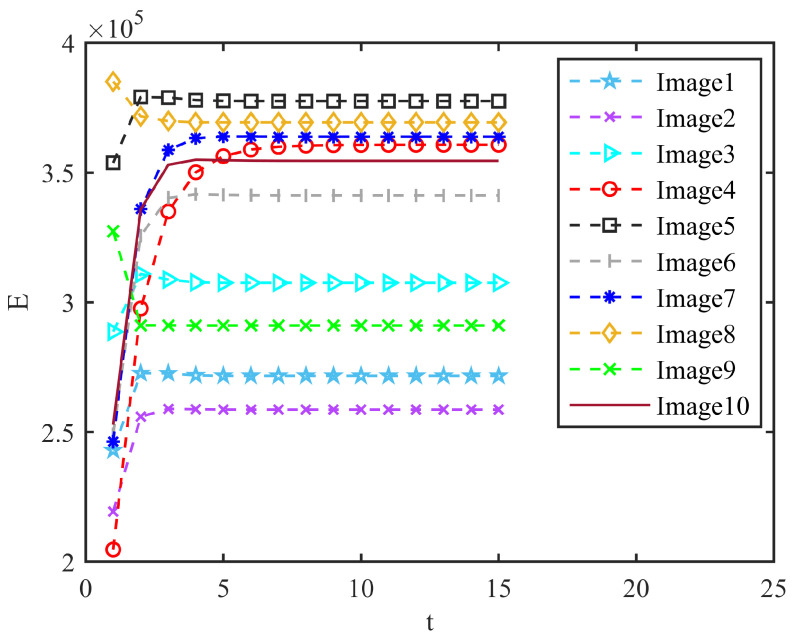
Value of E during CCNN iteration.

In order to study the statuscharacteristics of the CCNN, we measured the values of $Y=\frac{1}{1+e^{-(U_{ij}\left(n\right)-E_{ij}\left(n\right))}}$ and $E=e^{-\alpha_{e}}E_{ij}\left(n-1\right)+V_{E}Y_{ij}\left(n-1\right)$ at different iteration times. The results were as follows. t is the iteration number of the CCNN. It can be observed that the final steady-state iteration times of the different images were inconsistent during the iteration process, but generally, 6 to 7 times can be stable as it shows in Figure 9 and Figure 10.

**Figure 11:**
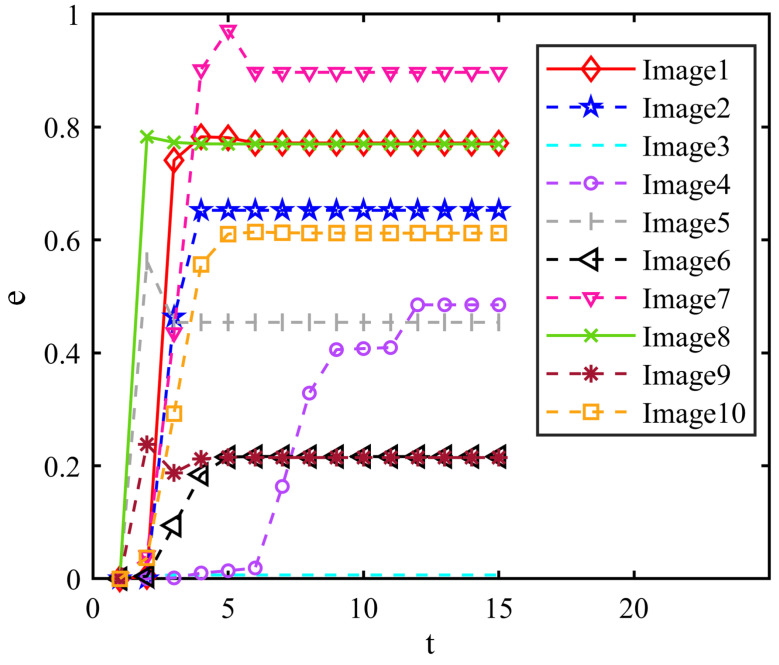
Value of e during CCNN iteration.

We used the change in the image entropy as the basis for judging whether to continue the iterations. From the results, we can find that the entropy of all images remained constant when the number of iterations was 15 as it shows in Figure 11.

## 5. Limitations and Future Work

Although this work provided a breakthrough for the task of detecting UAVs in infrared images, a few limitations remain, which future studies should address:

(1) The result showed that the model still had the phenomenon of missing detection. In the future, we hope to introduce structural information of the UAV to better detect all parts of the UAV.
(2) How to design an algorithm for video tasks based on the characteristics of the CCNN model to achieve the goal of real-time detection is also one of the important tasks in the future.
(3) In this work, we used the CCNN to process infrared images and realize the detection of UAVs. In the future, we will work on implementing hardware-based CCNNs based on memristive synaptic devices.

## 6. Conclusions

In this work, we designed an automatic detection framework for infrared UAV images based on the CCNN, which processes infrared images without learning, solving the problem in deep learning due to insufficient data. All calculated parameters were automatically acquired in self-adaptive ways. Our framework segments the infrared images with a low number of iterations and has high-performance indicators, which is of practical value for small target recognition in infrared images.

## Figures and Tables

**Table 1:** IoU of the above Images 1–10.

Image	CCNN	PCNN	FCM	Otsu	Iteration	Bimodal
1	0.7479	**0.8333** ^1^	0.6598	0.7882	0.7995	0.5961
2	0.6218	0.1870	0.6768	0.1978	0.5975	**0.6964** ^1^
3	**0.5983** ^1^	0.2755	0.0039	0.0000	0.0000	0.0000
4	**0.7936** ^1^	0.3722	0.0022	0.0163	0.6322	0.2717
5	**0.9701** ^1^	0.6494	0.0489	0.0000	0.0000	0.0000
6	**0.5576** ^1^	0.5330	0.3459	0.4425	0.4485	0.3125
7	**0.7865** ^1^	0.2043	0.0135	0.1218	0.1727	0.0000
8	**0.8654** ^1^	0.0002	0.3955	0.0000	0.0000	0.0000
9	0.7384	0.6638	0.2980	0.9353	**0.9782** ^1^	0.8000
10	**0.5843** ^1^	0.1384	0.0063	0.1568	0.1719	0.0000

^1^ Highest IoU among all methods.

## Data Availability

The data that support the findings of this study are available from the corresponding author upon reasonable request.
